# Operative Treatment of Pleuritic Effusions

**Published:** 1876-05

**Authors:** Herman Mynter


ART. III.—*Operative Treatment of Pleuritic Effusions* (*Serous*).

By HERMAN MYNTER, M. D. *

The theme I shall take the liberty of presenting to you this evening, and which I hope will be of some interest to you, although it may not offer anything new, is the operative treatment of the serous pleurisies. I have chosen this subject partly because I have a case to report, treated in this way, and partly because I am inclined to perform the operation at an earlier period than has been the custom, and believe that an early thoracentesis not only is allowed, but also from a pathologico-anatomical point of view is advisable.

I do not mention here the purulent pleurisies, empyema, because I am of the opinion that all agree to evacuate the matter here as soon as possible and only disagree as to the methods of doing it, although most physicians prefer a free incision.

An acute pleurisy commences like all inflammations with redness and hyperemia of the capilliary vessels, the epithelium is thrust off, the serosa losses its gloss and smoothness and a soft membranous exudate is built on the surface of the serosa. This exudate consists, according to Rindfleish, of blood fibrin from the capilliary vessels, and contains large numbers of the thrust-off epithelial cells. These epithelial cells are, according to the investiga-

*Read before the Buffalo Medical Association, Feb. 1, 1875.

tions of Rindfleish, capable of further development, and of forming tissue cells and vessels, and he is of the opinion, that most of the vessels afterwards found in this exudate, are formed of these epithelial cells, and only few come from the serosa. The more tissue there is formed, the more the fibrin vanishes by resorption, and while at first the membranous exudate could easily be removed from the serosa, the connection between them will now be more firm, the newly built tissue uniting with the tissue of the serosa. If the inflammation stops here we shall have a pleuritis sicca, and the serosa will have taken only a small part in the formation of the membranous exudate. Afterwards, we often find, that the pleura parietalis and viceralis have grown together, wholly or in part, on there is built a thick, fibrous, shining stratum, on different portions of the pleura. But generally the inflammation goes farther, and after some days the tissue of the serosa commences to take part iu the inflammation. The serosa will then be found filled with numerous young tissue cells, especially near the surface, and or the surface they form an independent stratum, a pseudo-membrane that has a tendency to organization and must be distinguished from the membranous exudate, formed of fibrin and epithelial cells. In this pseudo-membrane capilliary vessels are soon formed, and these capillary vessels are large and easily rupture, and we can thus get a hæmorrhagic exudate. While this membrane is being formed, a copious amount of serum is transuded from the vessels of the serosa which at first will be found in the lowest part of the cavity of the pleura, but, as it increases in quantity, it compresses the lung, displaces the liver, heart and diaphragm, expands the chest and inter-costal spaces.

If resorption takes place, the organs can and will return to their normal places, and the lung be perfectly expanded again. But according to Foster, this only takes place when resorption occurs in three or four weeks. If it lasts longer a change takes place that prevents the expansion of the lung and by that, complete recovery, and this is the retraction of the newly built tissue.

In accordance with the investigations of Brouardel the lung is surrounded after two or three weeks with a firm tissue-stroma, and by that prevented from perfectly expanding. The new building of tissue is not confined to the surface of the serosa but attacks the s erosa itself and the interlobular walls, and thns force its way into the lung.

Thus there is formed an interstitial pneumonia, and this commences during the first days of the inflammation. By that, too, the lung loses its ability of expansion, and when the resorption at last sets in, the thorax must sink in and we get deformity, scoliosis, with all its consequences. The retraction of the newly-built tissue in the interlobular walls has also another effect, and that is the dilatation of the bronchi, a relation that Niemeier has shown and that can produce other diseases.

But the resorption itself meets with difficulties, when the tissues commences to retract, the newly-built vessels, through which the resorption takes place, partly vanishing and partly getting smaller.

If we now perform thoracentesis at an early period, for instance in the first week, or as soon as the diagnosis is fixed, the relation will be modified. The retraction of the tissue has not commenced, and the expansion of the lung will take place in the same degree as the exudate gets smaller, and when this has gone, the lung is perfectly expanded, which we can show,

1st, By the difference in the percussion, which becomes clear, (generally it does not become perfectly clear, because the pseudo-membranes remain).

2d. By the respiration which is heard vesicular to the base of the lung.

3d. By the entrance of the vibrations of the voice, which, to be sure, are not felt quite as well as over the healthy lung, and

4th. By the frequent entrance of the friction sound; the patient too, feels much releived and breathes more easily and better.

But, you may say, you remove the exudate, but how will you prevent its coming again? It often comes again, but then you can operate again, and with a capillary trocar this operation is of so little importance that it hardly deserves the name of operation, and the danger is, judging by the numerous operations which have been performed, very small, if you are only careful to have a clean apparatus and to exclude the air.

Even if you penetrate the lung with such a small trocar, it will not be of great importance and will result only in a small interstitial pneumonia around the wound. But often the exudate does not appear again, and possibly the newly-built tissue has something to do with that. It is elastic, and when the lung now suddenly expands, the same change must occur in the tissue as when it retracts gradually, namely compression of the vessels, and so, what without thoracentesis prevents the resorption, might possibly after the thoracentesis prevent a new exudation. This is only my theory, but it is a fact, that the exudate often does not appear again, and that immediate recovery often occurs after a thoracentesis, performed at an early period. A danger that has been thought important is the possible transformation of a serous exudate into a purulent, that is, the formation of empyema. I do not remember to have seen that occur, although I have seen many early thoracenteses performed, and when the apparatus is clean, and the air excluded, I do not believe that the operation has to bear the blame if this occurs.

As to the early operation itself, experience has shown that the prospect of recovery diminishes in direct proportion to the duration of the disease. Thus Dr. Evans found, that the percentage of death, when the operation was performed in the fourth week was 12 per cent.; 23 per cent. when it was performed between the first and second month; 50 per cent. in the third month, and 62 per cent. after the fourth month. He has gathered 308 cases in which the operation was performed for serous exudate, and only in two cases death occurred owing to the operation, in one case through hæmorrhage from an artery wounded by the operation, and in another from the shock. And even in cases where death occurs afterwards, life was prolonged and the patient felt much relieved.

Another reason to operate early is, that pleuritis can occasion sudden death, through thrombosis of the pulmonary artery, produced by disturbances of the circulation in a mechanical manner. Moreover a compressed lung is especially exposed to tuberculosis and other chronic diseases, and for that reason an early operation would be advisable as the lung, after a late operation cannot be perfectly expanded again. Evans advises the early operation especially in cases where the exudate comes stealthily and without constitutional disturbances and is only discovered through physical examination, cases which especially defy medical treatment.

The operation is very easy. The patient is put in a half-sitting position and the thrusting-in is made in the posterior axillary line between the seventh and ninth ribs, and if possible, nearer the lower than the upper rib to avoid the artery.

It is not necessary to use chloroform as the operation is not very painful. Different instruments have been invented, which I shall not mention, but confine myself to the instrument we use in Denmark, and that unites moderate cost with solidity and simplicity. It consists of a trocar, united with a glass syringe by a rubber tube, on which we have a double cock. When the trocar has been introduced, and the stylet is to be taken out, the patient must stop breathing for a moment, till you have united the rubber tube with the trocar, that no air shall come by the inspiration, into the cavity of the pleura. For the rest, the apparatus explains itself.

When all fluid has been drawn out the little wound must be closed with a piece of plaster or collodium.

During the evacuation of the cavity, generally strong attacks of coughing come on which can be relieved by giving, before the operation, a subcutaneous injection of morphine. I remember also to have seen and read that a copious, thin, albuminous expectoration aften follows the operation, but shall not try to explain this phenomenon.

Lastly I will take the liberty to report a case I have had under treatment. The patient, Mr. Glass, forty-five years of age, has never before had any disease of the lungs. On the 27th of December, 1875, I saw him in his home after having for some time previously treated him for lumbago, and he complained then of pains in his right side, especially on deep inspirations, but as physical examination did not show anything abnormal and the patient had no fever, I considered the case to be pleurodynia, and treated it with derivatives. The pain continued however without change, he lost his appetite, felt thirst. but had very little fever, the temperature never being over 99° F.

On the third of January, 1876, distinct signs of pleurisy were first discovered. The percussion was dull, in front from fifth rib, behind almost from the angel of the scapula, the respiration and vibrations of voice were heard and felt weakened, no expectoration or sign of pneumonia. The pulse was 100, there was no increase of temperature.

During the following days the exudate increased, so that on the 9th of January it reached in front to the third rib, behind to the spine of the scapula, and the respiration was inaudible over the dull parts. The general health had grown worse, he did not take any nourishment, was very short of breath and uneasy. I therefore performed—Dr. Hopkins being present—thoracentesis, by which fully two quarts of sero-fibrinous, slightly hæmorrhagic exudate was removed. Immediately after the operation, the percussion became almost clear and the respiration was heard vesicular to the base of the lung; the vibrations of the voice also, could now be felt, the patient felt much relieved and breathed more easily.

The following days the exudate increased again, but only in a very little degree, so that the percussion never was perfectly dull to the angle of the scapula and so that the respiration and the vibrations were heard and felt to the base of the lung, but not so distinct as on the left side.

He commenced to have appetite, could sleep during the nights, the pain in the side decreased, especially after the application of a vesicatory, and he gained strength every day. From the 14th of January the dullness on percussion commenced gradually to decrease again, the respiration was heard more and more distinctly, and on the 22d of January he was able to leave the bed, all signs of pleurisy having almost disappeared.

We have then here a case of acute serous pleurisy, that came stealthily and only was discovered by the physical examination, a case that probably would have taken many weeks, if not months, for resorption without operation, even supposing this could have been accomplished.

The operation was performed on the sixth day of the disease, and the case terminated in almost immediate recovery.

I have often seen the same result following this operation, and never saw any bad effect of the operation, and I therefore feel inclined to operate early and believe, the earlier the better.

I do not believe you can operate too early, but you may operate too late.

Before the publication of this paper another case of pleuresy occurred in my practice which I treated in the same way, and reported in the April meeting, and as this case, in my opinion, especially shows the value of the thoracentesis as a curative operation, I will take the liberty to add it here. The patient, Mr. Casey, 39 years of age, consulted me on the 10th of March, 1876. He has always enjoyed good health and does not think he has ever been sick. His disease commenced about twenty days previously with pain and stitch in the left side, a little cough with bronchitic expectoration, some shortening of breath and with only little fever, so that he had not been obliged at all to keep his bed. The pain in the side decreased gradually, but the shortening of breath increased slowly, but so much but that he, on the 10th of March, was able to walk from his house to me and back again, a distance of more than two miles.

At the examination I found dull percussion, behind from the midst of the regio infraspinata, in front from fifth rib, and slight tympanitic percussion below the collar-bone.

The respirations and vibrations of voice were heard and felt much weakened, almost inaudible, over the dull parts. He had no expectoration, no rales were heard, he made thirty-two inspirations in a minute. I advised him to have thoracentesis performed but he would not consent. After having, for the following six days, been treated by another physician, who advised him by no means to have this operation performed, he came back to me on the 16th of March, 1876, and declared he should have the operation performed as he felt worse and worse, but still that day he was able to walk, in very bad weather, to me and back again.

The shortening of breath had increased a little, he made thirty-six inspirations in a minute, but the percussion was now dull behind to the spine of the scapula, in front to the second rib, and the respirations and vibrations of voice were perfectly inaudible over the dull parts. The pulse was 100, the temperature 98°, the heart displaced. The next day, the 17th of March, I performed thoracentesis—Drs. Hopkins and Fowler being present—by which fully three quarts and a half i. e. 3500 cub. cent. sero-fibrinous exudate were removed. The exudate coagulated immediately after the removal. He felt better after the operation, the percussion became perfectly clear in front, and almost clear behind, and the respirations were heard to the base of the lung, but a little weakened, the vibrations of voice were also felt, but weakened.

He coughed some during the following night, but except that he felt comfortable. The next day, the 18th of March, he made only twenty inspirations in a minute, he had no fever, the pulse was 96, the percussion and the respirations as the day before, the heart in its right place. A soft friction sound was heard for some days. From the 18th of March to the 31st of March, on which day he was able to leave his bed, he gained strength rapidly; he had not a single time fever, the temperature never being over 98°, the cough ceased gradually, the percussion became almost clear in the regio infrascapularis, the respirations and vibrations of voice became nearer and nearer normal, and on the 31st of March the only sign of his disease was a slight dullness in the lower part of the regio infrascapularis.

In my opinion, this case shows that this operation is, or at any rate can be, curative, and I myself have not the slightest doubt that I have cured the patient by aid of the operation. I cannot deny that he might have recovered without the operation, but it would by such a copious and still increasing exudate at any rate have taken months, and by the operation he recovered in fourteen days, and avoided all the dangers that follow this disease when it is protracted.

The result in these two cases is at any rate encouraging, and I myself will perform the operation as often as I get an opportunity.

